# Supplementary figures and images for: Long-term effects of straw and straw-derived biochar on soil aggregation and fungal community in a rice–wheat rotation system
Source: PeerJ. 2019 Jan 4;6:e6171. doi: 10.7717/peerj.6171 (PMC6322488; doi:10.7717/peerj.6171)

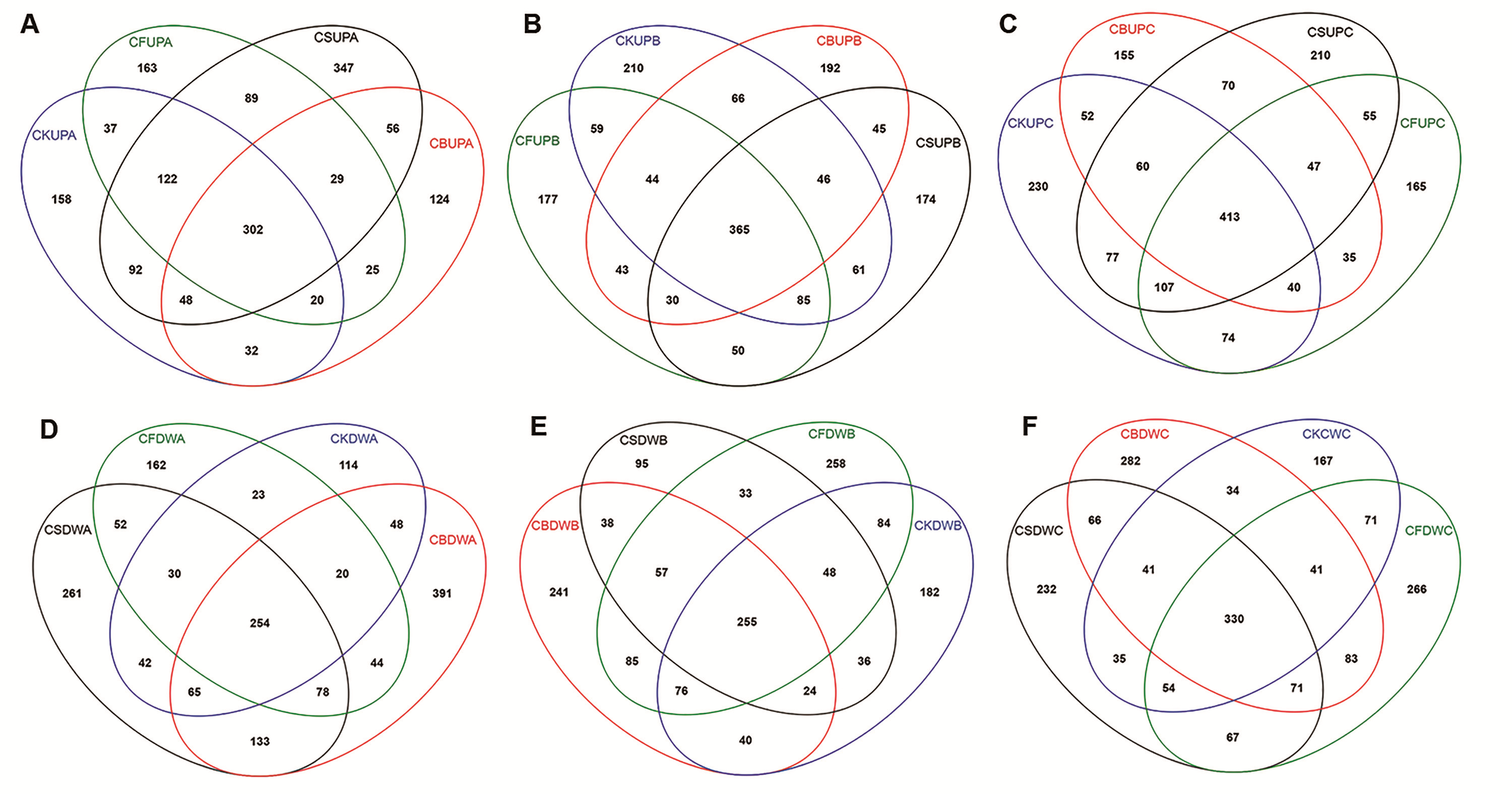

Supplement: Supplemental Information 1 — Among the group names, “A,” “B,” and “C” refer to macroaggregate, microaggregate, and silt clay, respectively. [file peerj-07-6171-s001.png]

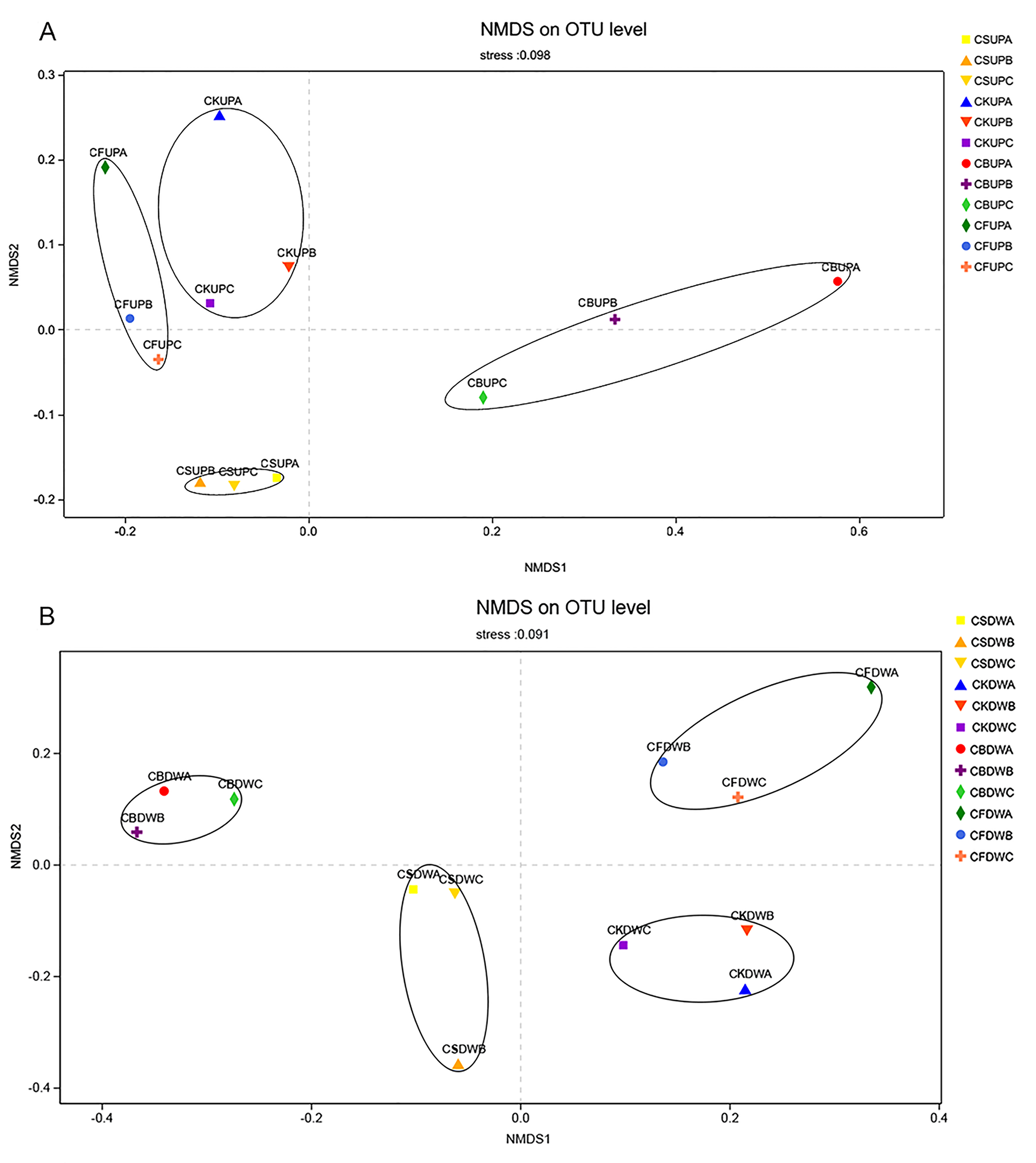

Supplement: Supplemental Information 2 — “UP” and “DW” referred to the soil depths of 0–20 and 20–40 cm, respectively. “A,” “B,” and “C” meant macroaggregate, microaggregate, and silt clay, respectively. [file peerj-07-6171-s002.png]

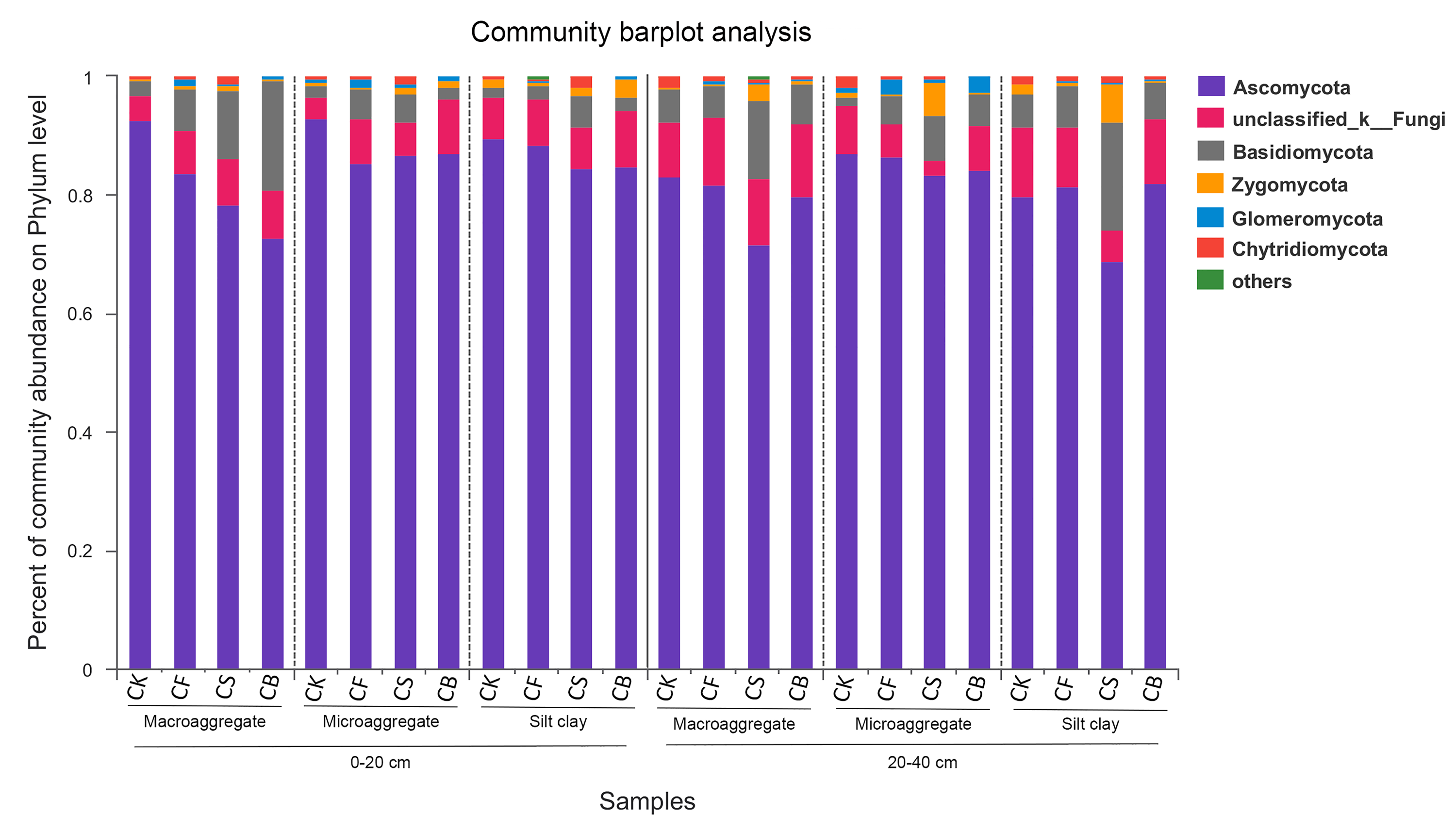

Supplement: Supplemental Information 3 — Relative abundances are based on the proportional frequencies of the DNA sequences that could be classified. [file peerj-07-6171-s003.png]
