# Supplementary material for: Long-term effects of straw and straw-derived biochar on soil aggregation and fungal community in a rice–wheat rotation system
Source: PeerJ. 2019 Jan 4;6:e6171. doi: 10.7717/peerj.6171 (PMC6322488; doi:10.7717/peerj.6171)
Supplement: Supplemental Information 4 — The characteristics of straw and biochar listed were the average values for 6 years. [file peerj-07-6171-s004.docx]

| Items | Rice straw | Rice straw biochar | Wheat straw | Wheat straw biochar |
| --- | --- | --- | --- | --- |
| TN (g kg^-1^) | 3.84 | 17.10 | 3.30 | 8.40 |
| TP (g kg^-1^) | 1.05 | 2.79 | 0.83 | 2.27 |
| TK (g kg^-1^) | 15.27 | 39.17 | 11.20 | 43.98 |
| Ash content (%) | _ | 37.40 | _ | 20.80 |
